# Oxidative Stress and Reprogramming of Lipid Metabolism in Cancers

**DOI:** 10.3390/antiox14020201

**Published:** 2025-02-10

**Authors:** Siqi Li, Hang Yuan, Liang Li, Qin Li, Ping Lin, Kai Li

**Affiliations:** Division of Abdominal Tumor Multimodality Treatment, Cancer Center and Lab of Experimental Oncology, State Key Laboratory of Biotherapy, West China Hospital, Sichuan University, Chengdu 610041, China; siqili@stu.scu.edu.cn (S.L.); yuanhang@wchscu.edu.cn (H.Y.); liliang@wchscu.edu.cn (L.L.); hxliqin@wchscu.edu.cn (Q.L.)

**Keywords:** redox signaling, oxidative stress, lipid metabolism, cancer, biomarker, cancer therapy

## Abstract

Oxidative stress is a common event involved in cancer pathophysiology, frequently accompanied by unique lipid metabolic reprogramming phenomena. Oxidative stress is caused mainly by an imbalance between the production of reactive oxygen species (ROS) and the antioxidant system in cancer cells. Emerging evidence has reported that oxidative stress regulates the expression and activity of lipid metabolism-related enzymes, leading to the alteration of cellular lipid metabolism; this involves a significant increase in fatty acid synthesis and a shift in the way in which lipids are taken up and utilized. The dysregulation of lipid metabolism provides abundant intermediates to synthesize biological macromolecules for the rapid proliferation of cancer cells; moreover, it contributes to the maintenance of intracellular redox homeostasis by producing a variety of reducing agents. Moreover, lipid derivatives and metabolites play critical roles in signal transduction within cancer cells and in the tumor microenvironment that evades immune destruction and facilitates tumor invasion and metastasis. These findings suggest a close relationship between oxidative stress and lipid metabolism during the malignant progression of cancers. This review focuses on the crosstalk between the redox system and lipid metabolic reprogramming, which provides an in-depth insight into the modulation of ROS on lipid metabolic reprogramming in cancers and discusses potential strategies for targeting lipid metabolism for cancer therapy.

## 1. Introduction

Redox signaling is an important intracellular signaling mechanism. The metabolism of cells provides the material and energy basis for all life activities of living organisms. Redox reactions—i.e., the gain, loss, and transfer of electrons—are key components in the cellular metabolic network [1]. In solid tumors, the rapid growth of tumor tissues, accompanied by a lack of supply from the vascular system, leads to an insufficient supply of oxygen and nutrients within the tumor tissues; here, the tumor microenvironment (TME) presents with overall hypoxia. However, hypoxic conditions are conducive to increasing reactive oxygen species (ROS) levels and oxidative stress. Under hypoxic conditions, cancer cells preferentially choose aerobic glycolysis to provide energy and biosynthetic precursors for their rapid proliferation, which is called the Warburg effect. The process of massive aerobic glycolysis produces more substances, such as ROS, which increases the level of intracellular oxidative stress [2]. This oxidative stress can further induce the activation of relevant signaling pathways and alterations in gene expression, promoting the survival, proliferation, invasion, and metastasis of cancer cells. On the other hand, high levels of oxidative stress interact with and counterbalance cellular metabolism, such as lipid homeostasis. Lipid metabolism, an essential physiological process, is aberrantly activated in tumors due to enhanced lipid synthesis or uptake, contributing to rapid cancer cell growth and tumor formation [3], and lipid metabolism reprogramming is also an important characteristic of cancer. Both oxidative stress and lipid metabolic reprogramming are considered important cancer therapeutic targets, and their related mechanisms constitute important research directions in the fields of cell biology and pharmacy. In this review, we discuss the connection between oxidative stress and lipid metabolic reprogramming in cancer cells and how these two pathways affect tumor development from the perspective of the redox system and metabolism regulation. Finally, we explore the development prospects of targeted lipid metabolism therapy for cancer on the basis of relevant clinical studies in recent years.

## 2. Oxidative Stress in Cancer Cells

In redox reactions, electrons flow from the reducing agent to the oxidizing agent. The three major intracellular redox pairs—nicotinamide adenine dinucleotide hydrogen/nicotinamide adenine dinucleotide (NADH/NAD^+^), nicotinamide adenine dinucleotide phosphate hydrogen/nicotinamide adenine dinucleotide phosphate (NADPH/NADP^+^), and glutathione/glutathione disulfide (GSH/GSSG)—are responsible for the majority of cellular electron transfer. These redox pairs are used as cofactors or substrates to neutralize ROS in enzymatic or non-enzymatic reactions to maintain intracellular redox homeostasis [4]. In addition, a variety of redox-related substances exist in the cell to mediate redox signaling, including small molecules and protein macromolecules. The oxidative system mainly consists of reactive oxygen species (ROS), reactive nitrogen species (RNS), reactive sulfur species (RSS), and other reactive molecules. Among them, reactive oxygen species (ROS) include free radical species such as superoxide anion (O_2_^•−^), hydroxyl radical (^•^OH), alkoxy radical (^•^OOR), and peroxide radical (^•^OOH), as well as non-free radical species such as hydrogen peroxide (H_2_O_2_) and singlet oxygen (^1^O_2_) [5]. Reactive nitrogen species (RNS) include nitric oxide (NO), nitrogen dioxide (NO_2_), peroxynitrite (ONOO^−^), and nitroxyl (HNO) [6]. Reactive sulfur species (RSS) mainly include hydrogen sulfide (H_2_S) and sulfur dioxide (SO_2_) [7]. The antioxidant system mainly includes large-molecule antioxidant enzymes such as superoxide dismutase (SOD), catalase (CAT), and glutathione peroxidase (GSH-Px), as well as small-molecule antioxidants such as glutathione, vitamin C, vitamin E, and coenzyme Q10 [8]. In addition, glutathione (GRX), peroxiredoxin (PRDX), thioredoxin (TRX), and thioredoxin reductase (TRXR) are also important intracellular antioxidants that form the cellular antioxidant defense system together with NADPH through synergistic action [9]. Cells use these molecules and regulatory networks to respond to basal signaling and overaccumulation of oxidative stress and maintain redox homeostasis.

**Figure 1:**
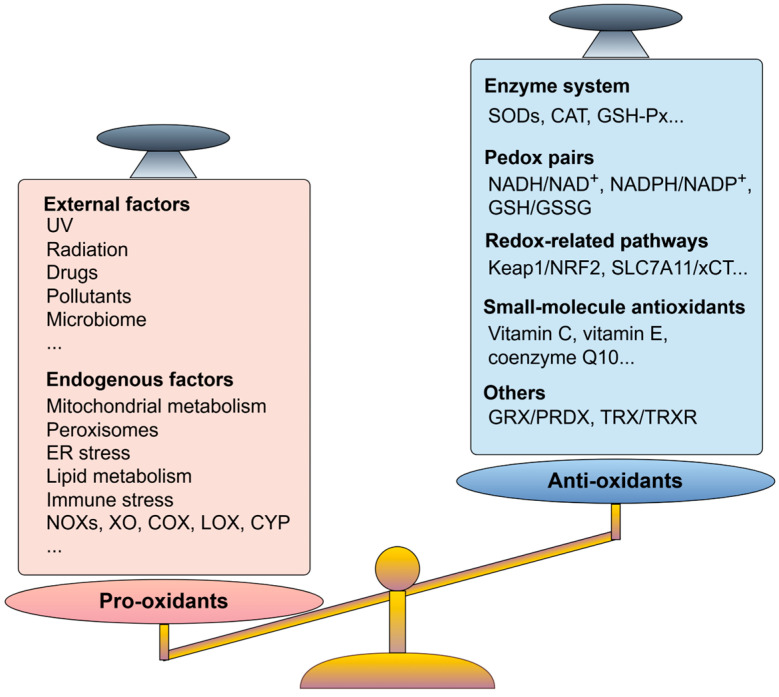
Pro-oxidants and anti-oxidants that maintain redox homeostasis in cells. Under normal physiological conditions, basic redox metabolism occurs in the body, and the oxidative and antioxidant capacities are in balance. When the oxidative system and antioxidant system are out of balance, cells experience stress and dysfunction.

Under normal physiological conditions, basic redox metabolism occurs in the body, and the oxidative and antioxidant capacities are in balance. When the oxidative system and antioxidant system are out of balance, cells experience stress and dysfunction, which is called oxidative stress (Figure 1). Accumulating evidence reports that ROS have dual roles as signaling molecules involved in intracellular signal transduction at low–moderate levels, such as influencing cellular metabolism and regulating gene expression. In contrast, overproduced ROS can attack lipids, proteins, and nucleic acids in the cell, resulting in oxidative damage to molecules; sustained oxidative damage leads to organismal aging and pathology, such as neurodegenerative diseases, diabetes, atherosclerosis, and cancers [10,11,12,13]. Therefore, redox homeostasis is essential for maintaining a normal physiological environment.

Alterations in redox homeostasis during tumor development in response to high levels of ROS manifest in the following ways: the high metabolic levels required for tumor growth increase the need for electron acceptors such as NAD^+^; the active synthesis and metabolism of cancer cells increase the need for the reducing power required for biomolecule synthesis; and features of the tumor microenvironment lead to increased antioxidant defenses, promoting cancer cell survival. These alterations in redox homeostasis result in higher levels of oxidative stress in tumors in comparison with the normal physiological environment, which stimulate tumor growth, invasion, and metastasis. Importantly, to avoid excessive oxidative stress-induced cell death, cancer cells adapt to oxidative stress in multiple ways. For example, the key cellular redox sensor transcription factor NFE2-related factor 2 (NRF2) activates the transcription of antioxidant genes—including quinone oxidoreductases (NQO1) and heme oxygenase-1 (HO-1)—to prevent oxidative stress-induced cell death [14]. NRF2 participates in the regulation of oxidant-stimulated programmatic functions, including autophagy, inflammasome assembly, ER stress/UPR, mitochondrial biogenesis, and stem cell regulation [15]. The effect of NRF2 can be bidirectional, and great progress has been made in the clinical development of its activators. Sulforaphane has the potential to prevent the toxic and neoplastic effects of environmental carcinogens, as well as to ameliorate a diversity of conditions characterized by chronic oxidative, metabolic, and inflammatory stress [16,17]. NRF2 activation due to KEAP1 or NRF2 mutations occurs frequently in many cancers, suggesting that the inhibition of NRF2 may be a promising therapeutic strategy. The NRF2 inhibitor clobetasol propionate was found to induce oxidative stress and strongly inhibit the growth of lung cancer cells with the KEAP1 mutant [18]. Cancer cells can also produce a variety of antioxidants (such as NADPH and GSH) via the activation of adenylate-activated protein kinase (AMPK), the pentose phosphate pathway (PPP), and a reduction in glutamine and folate metabolism [19]. Our previous study demonstrated that ZNF32 contributed to anoikis resistance by maintaining redox homeostasis and activating Src/FAK signaling in hepatocellular carcinoma [20]. In addition, the microenvironment of cells and other external factors can also affect the fate of tumor cells under the action of ROS. For example, interactions with surrounding cells, nutrient supply, etc., may alter the sensitivity and coping strategies of tumor cells in relation to ROS. However, when cancer cells are subjected to excessive oxidative stress above the critical threshold, cancer cells are unable to repair oxidative damage, and cell death occurs. Thus, pro-oxidant-based anticancer therapy becomes an effective and promising strategy. Piperlongumine has been well characterized to selectively kill cancer cells by potently increasing ROS generation [21]. Our recent study revealed that alkannin-induced cytotoxic autophagy and apoptosis by promoting ROS-mediated mitochondrial dysfunction and activation of the JNK pathway [22]. In conclusion, the fate of tumor cells in response to ROS is determined by a variety of factors, including the level of ROS, the metabolic cell’s own antioxidant capacity and survival mechanisms, and the external environment. These factors interact to dynamically affect the death or survival of tumor cells.

### 2.1. The Formation of ROS

In vivo, ROS production is induced mainly by intracellular biological processes; the main endogenous sources of ROS include mitochondria, endoplasmic reticulum (ER), peroxisomes, and enzymatic reactions. During the aerobic respiration of mitochondria, the electron transport chain produces ROS as byproducts, which are partially reduced to O_2_^•−^ when electrons are transferred to O_2_. Mitochondrial complexes I and III produce O_2_^•−^, which is the most abundant ROS in the cell and can be converted to H_2_O_2_ by superoxide dismutase (SOD) [23]. Although it is not as reactive as other ROS, H_2_O_2_ can easily diffuse across the biofilm to damage nearby cells. Through an iron-dependent Fenton reaction, H_2_O_2_ produces ^•^OH [24], which is highly reactive and can damage lipids, proteins, and DNA. In addition, O_2•_ can react with NO to form peroxynitrite (ONOO^−^), a highly reactive nitrogen substance (RNS) that can cause lipid and DNA damage [6]. The ER lumen is an oxidative environment that promotes the biochemical reactions required for protein folding. Endoplasmic reticulum oxidoreductase (ERO-1α) releases H_2_O_2_ to promote the oxidation of ER proteins during the production of disulfide bonds [25]. Like mitochondria, peroxisomes are also high consumers of oxygen; although they do not produce ATP, they produce ROS, mainly H_2_O_2_. The NADPH oxidase family (NOX), located in the cytoplasmic membrane, transfers electrons from intracellular NADPH to extracellular oxygen molecules to reduce them, producing O_2_^•−^ or H_2_O_2_ [26]. ROS are also generated during immune responses, such as the phagocytosis of pathogens by macrophages and neutrophils, which directly destroy pathogens by generating large amounts of ROS—this is a specific physiological process known as the “respiratory burst”. NOX plays a key role in this process and plays a role in killing pathogens by producing bactericidal reactive oxygen species such as H_2_O_2_ through redox metabolism [27]. Other enzymatic reactions, such as those involving xanthine oxidase (XO), cyclooxygenase (COX), lipoxygenase (LOX), cytochrome P450 enzyme (CYP), and catalase (CAT), also produce ROS as byproducts, resulting in subsequent peroxidation or *β* oxidation of fatty acids [23,28]. In addition to endogenous factors, external stimuli such as ultraviolet irradiation, radiation, drugs, and pollutants can also promote the production of ROS in cells. In addition, microbes can also promote ROS production through a variety of mechanisms, thereby affecting the health status of the host. These microorganisms include bacteria, viruses, fungi, etc., which activate the host’s immune system through direct or indirect ways, resulting in the generation of ROS; they may have an important impact on the occurrence and development of diseases and cancers. An integrative multi-omics study highlights that oxidative-stress-related genes leading to Crohn’s disease are regulated by DNA methylation and host–gut microbiota interactions [29]. Helicobacter pylori mediates chronic inflammation of host cells and subsequently induces DNA methylation, leading to cell proliferation and evolution into poorly differentiated gastric cells, which provides the pathological basis for the occurrence of advanced gastric cancer [30]. Oxidative-stress-related genes can be affected by environmental factors, inflammation, microbiota, and epigenetic changes, influencing the progression of disease or cancer. Thus, ROS and RNS constitute a large and important population of active reactants that play a key role in intracellular biological processes.

### 2.2. The Control of ROS

Intracellular ROS are important signaling molecules, and these ROS signals can promote or inhibit tumor growth or metastasis under different circumstances. ROS are the major intracellular signal transducers that maintain autophagy [31]; ROS modulate epigenetic regulation of gene expression by altering DNA methyltransferase (DNMT) or histone deacetylase (HDAC) activity [32]; and ROS such as H_2_O_2_ can reversibly oxidize cysteine residues on target proteins. These oxidative post-translational modifications (PTMs) control the biological activities of many enzymes and transcription factors and affect their functions [33]. In addition, ROS accumulation can cause senescence and several forms of cell death, including ferroptosis [34]. In cancers, the increase in membrane and mitochondrial production due to proliferation, as well as alterations in the antioxidant system, can cause changes in the ROS content. Depending on the metabolic properties of different cells and stimulatory signals from different growth factors or chemokines, many factors can also trigger a physiological increase in ROS. Under normal physiological conditions, ROS, similar to other signaling molecules, are produced at low levels and are restricted to subcellular localization, inducing signaling pathways to maintain normal physiological processes [35]. The concentration of ROS is tightly regulated within cells because high concentrations of ROS can cause nonspecific oxidation of targets, damage macromolecules, cause cellular dysfunction, and trigger stress response mechanisms such as inflammation or cell death.

#### 2.2.1. ROS Removal

The ways in which cells respond to ROS can be summarized as elimination and regulation. First, the intracellular antioxidant system plays an important role in clearing and balancing ROS. SOD is the first line of defense against reactive oxygen species by neutralizing O_2_^•−^, the main components of which are the three SODs: SOD1, SOD2, and SOD3. SOD reduces the level of intracellular free radicals by participating in the clearance of intracellular O_2_^•−^ and catalyzing its conversion to hydrogen peroxide (H_2_O_2_) and O_2_. H_2_O_2_ is more stable than O_2_^•−^, but H_2_O_2_ can penetrate most cell membranes to further catalyze oxidation reactions and is still a toxic oxidant in vivo. CAT can promote the decomposition of H_2_O_2_ into O_2_ and H_2_O, protect cells from H_2_O_2_ toxicity, and play a role in scavenging ROS. CAT is present in peroxisomes, the cytoplasm, and mitochondria. Unlike CAT, SOD is a strict subtype that is dependent on subcellular localization. SOD1 is localized in the intermembrane space (IMS) of the cytoplasm and mitochondria, SOD2 is present only in the mitochondrial matrix, and SOD3 is present only in the extracellular matrix [36]. The subcellular localization of SOD suggests that ROS are tightly controlled in different regions and that SOD-regulated changes in H_2_O_2_ concentration also promote H_2_O_2_ flux generation and signal transmission. To maintain ROS homeostasis, cells have a variety of antioxidant systems that play a role in scavenging ROS. In addition to CAT, PRDX and GSH-Px are important components of the cellular antioxidant defense system, and the cofactors for the catalytic reactions in both systems are TRX and reduced GSH, respectively. They are also utilized by glutathione-s-transferases (GSTs) to detoxify oxidative-stress-generated reactive compounds [9,37]. Notably, all these processes require the collaboration of NADPH. In addition to ROS detoxification, NADPH is required for many other cellular processes, including driving anabolic reactions such as those involving fatty acids and nucleotides; NOXs also use NADPH to generate ROS [26]. Therefore, maintaining NADPH homeostasis is critical for different intracellular biological processes. Other antioxidants, such as coenzyme Q10, vitamin C, vitamin E, and other molecules, also play important antioxidant roles in the response to ROS. Many small-molecule antioxidants, such as vitamin C, vitamin E, and carotenoids, chelate metal ions to reduce their catalytic activity, producing ROS [38].

In addition to traditional antioxidant systems, ROS production in mitochondria, called mtROS, can be limited by autophagy processes. Abnormal expression of mitochondria-related proteins in cancer cells leads to increased ROS production in the mitochondrial respiratory chain. Metastatic and spreading cancer cells promote mitophagy to remove damaged mitochondria and limit ROS production, thereby maintaining the survival of cancer cells [39].

#### 2.2.2. Spatial Regulation of ROS

The spatial regulation of ROS mainly emphasizes the specific response and management mechanism of ROS in different regions of the cell, and this regulation is based on the regulation of ROS by antioxidants. The scavenging of ROS by antioxidants is a widespread and direct intracellular coping strategy that reduces the level and harm of ROS by chemically reacting with them and converting them into more stable substances. The effects of antioxidants are often not confined to specific spatial locations but rather act throughout the cellular environment to maintain the overall stability of intracellular redox homeostasis. The spatial regulation of ROS focuses on the precise modulation of ROS production, distribution, and action at specific spatial locations.

Spatial regulation helps maintain redox homeostasis in specific regions of the cell. Since different cellular compartments have unique functional and metabolic requirements, precise spatial regulation ensures that ROS act in the right place; accordingly, they are able to prevent nonspecific damage caused by ROS to key biomolecules or excessive damage to other sensitive regions. Different studies have also summarized and described the local roles of ROS on the basis of differently localized ROS. For example, the Fenton reaction between fatty acids and iron ions in cells generates lipid reactive oxygen species (lipid ROS); here, SLC7A11/xCT transports cystine from the extracellular to the intracellular to be used as a raw material to make the antioxidant GSH. Then, GPX4 scavenges lipid ROS to avoid damage to the cell membrane by consuming GSH [40]. Moreover, certain organelles may regulate reactive oxygen species in a targeted manner according to their functions and needs for localized signal transduction or regulation of specific physiological processes. This way is much more specific and local, and it is a relatively fine regulation mode. In the process of tumor metastasis, tumor migration is directed according to external chemical signals. The generation of a ROS concentration gradient caused by uncontrolled mtROS can further drive the invasion and migration of cancer cells [41]. Through relevant regulation, ROS production and clearance can be restricted to specific regions to avoid inappropriate ROS distribution that may trigger extensive damage. An example of this is the strict regional expression of SODs and the distinct subcellular localization shown by the antioxidant PRDX and GPX isoforms [42]; other ROS-regulating enzymes in organelles, such as mitochondria and peroxisomes, can be used to illustrate the importance of the spatial regulation of ROS. In addition, there are differences in the tolerance and demand for ROS in different tissues and organs. The brain is one of the most sensitive organs to oxidative stress [43]. Oxidative stress is also a major factor in the abnormal function of the lung; this is because the lung organ is constantly exposed to air—it needs a stronger antioxidant defense system than other tissues do to prevent a range of possible damage caused by exposure to oxidants [44]. Spatial regulation allows the ROS content to adapt to the physiological characteristics and functional needs of this tissue to safeguard overall physiological coordination. In general, spatial regulation and antioxidant clearance complement each other to ensure the ability of cells to cope with reactive oxygen species and maintain normal physiological functions.

## 3. Reprogramming of Lipid Metabolism in Cancer Cells

The metabolic characteristics of cancer cells are significantly different from those of normal cells. Although cancer cells are highly metabolically heterogeneous, they require excessive amounts of lipids to maintain constant cell proliferation and adapt to the living environment. Therefore, lipid metabolism reprogramming, including the abnormal metabolism of fatty acids, phospholipids, and cholesterol, is one of the most prominent metabolic alterations in cancer [3]. Lipid metabolism is an important biological process in cells. Lipid metabolism converts nutrients into metabolic intermediates for membrane biosynthesis, energy storage, and the production of signaling molecules. Cancer cells can fulfill lipid and energy requirements by activating de novo lipogenesis (DNL), increasing fatty acid (FA) uptake, and promoting fatty acid oxidation (FAO). Owing to their high metabolic and anabolic stress, normal cells obtain lipids through both food intake and hepatic synthesis, and cancer cells are more inclined to activate adipogenesis to circumvent limited lipid uptake. As early as the 1980s, researchers found that almost all FAs in cancer cells were produced by de novo synthesis [45], suggesting that FA synthesis is an important cellular process in tumorigenesis and development.

Lipids are hydrophobic biomolecules that include mainly fatty acids, sterol lipids, glycerolipids, phospholipids, sphingolipids, and saccharolipids. These lipids are components of biological membranes, are used for energy metabolism and storage, and play important roles as signaling molecules. Fatty acids are a variety of molecules composed of hydrocarbon chains of different lengths and degrees of unsaturation, and many lipids are synthesized from FAs [46]. Most FAs can be synthesized intracellularly or ingested through food. The majority of lipid molecules in the human diet are triacylglycerols (TAGs) and cholesterol. Other essential fatty acids, such as linoleic acid (LA), α-linoleic acid (ALA), and other polyunsaturated fatty acids (PUFAs), must be ingested through food and generally exist in foods such as vegetable oils and nuts. FAs constitute the hydrophobic tail of phospholipids and, together with cholesterol and a small number of glycolipids, constitute the major component of biofilms. FAs can also be assembled into TAGs, which are synthesized and stored when nutrients are abundant, releasing large amounts of energy during catabolism [47]. Most cells are able to store triglycerides to some extent in intracellular organelles called lipid droplets. Lipid droplets maintain lipid balance, prevent lipid toxicity, and produce ATP by decomposing the lipids stored in the lipid droplet under conditions of metabolic stress. Lipid homeostasis is regulated by compensatory and negative feedback in each process of lipid metabolism. The processes of lipid metabolism regulate lipid homeostasis through a combination of compensation and negative feedback. In addition to their role in energy storage, some lipids act as signaling molecules and hormones in a variety of cellular functions, such as signal transduction. For example, the hydrolysis of glycerolipids and sphingomyelins can also produce second messengers such as diacylglycerol (DAG), phosphatidic acid (PA), and phosphoinositol compounds, which transmit signals that enable cells to respond quickly to a stimulus [48].

The DNL process is the main source of lipids in cells. Cells primarily use pyruvate, the product of glycolysis, to supply the mitochondrial tricarboxylic acid cycle (TCA), and this process results in the production of citrate, which is cleaved in the cytoplasm by ATP citrate lyase (ACLY) to acetyl-CoA and oxoacetate. In addition, acetate conversion by acetyl-CoA synthetase (ACSS) is another pathway for acetyl-CoA production by DNL. CoA is activated by acetyl-coenzyme A carboxylases (ACCs) to produce malonyl-CoA, which is then catalyzed by fatty acid synthase (FASN) to produce a saturated fatty acid (SFA)—palmitic acid (PA, C16:0), which is the initial product of FA synthesis. Palmitic acid can be lengthened by elongase of very-long-chain fatty acids (ELOVLs) to produce molecules of different lengths and saturations. SFAs can be desaturated by either stearoyl coenzyme A desaturases (SCDs) or fatty acid desaturases (FADSs) to synthesize monounsaturated fatty acids (MUFAs), such as oleic acid (OA, C18:1) and palmitoleic acid (C16:1). Furthermore, desaturations caused by ELOVLs and FADSs can convert ingested PUFAs, such as linoleic acid (LA, C18:2) and α-linolenic acid (ALA, C18:3), into other PUFAs, such as arachidonic acid (AA, C20:4) and adrenal acid (AdA, C22:4) [49,50]. The lipids involved in cellular biological processes are numerous and are constantly changing under conditions of different physiological, pathological, and environmental stimuli. Lipid metabolism reactions involve many reactions of enzymes and substrates and feedback, and key enzymes involved in lipogenesis are tightly regulated by transcriptional and post-translational modifications of different environmental factors. Mutation of key enzymes is an essential element in the metabolic reprogramming of cancer cells.

In addition to the endogenous DNL pathway, cells replenish the intracellular lipid pool via the uptake of exogenous lipids from the microenvironment under normal nutritional circumstances. Exogenous lipids can enter the cell either by passive diffusion or through the involvement of transporter molecules, including lipid translocases CD36, the fatty acid transporter protein family (FATP), and fatty acid binding proteins (FABPs) [51]. The increased demand for lipids in tumors is inevitably accompanied by an elevated capacity to take up lipids. The expression of FATPs is increased in a variety of cancers, such as melanoma, breast cancer, prostate cancer, and hepatocellular carcinoma, and promotes tumor growth through lipid metabolism reprogramming [52,53]. FABPs promote cervical cancer metastasis by increasing intracellular FA levels [54]; FABPs also play important roles in glioblastoma development [55]. ACSSs are important members of mammalian cells that uptake acetate and convert it into lipids and acetylate histones. ACSSs are highly expressed in a variety of cancer cells and maintain cancer cell growth in the absence of nutrient deficiency by catalyzing acetate [56].

FAO refers to the process in which glycerol and FAs produced by lipid hydrolysis are further oxidized and decomposed into CO_2_ and H_2_O_2_, releasing a large amount of energy to feed the organism. FAO occurs mainly in mitochondria. While short-chain and medium-chain fatty acids can enter mitochondria directly, long-chain FAs are transported by the carnitine shuttle system. First, long-chain FAs need to be covalently modified by acyl-CoA synthetase to form acyl-CoA, which cannot directly cross the inner membrane of mitochondria and needs to enter mitochondria through the carnitine shuttle system. In this process, acyl-CoA binds to carnitine and is converted to acylcarnitine by carnitine acyltransferase (CPT) so that it can cross the mitochondrial membrane via carnitine-acylcarnitine translocase (CACT) and enter the matrix of the mitochondria for *β*-oxidation. Within the mitochondria, *β*-oxidation forms CoA and further passes through the TCA cycle to produce NADH and FADH2, which are transferred to the respiratory chain for oxidative phosphorylation to generate ATP [57]. Mitochondria play a central role in intracellular lipid metabolism. In addition to this mitochondria-associated regulation, fatty acid synthesis and oxidation are also regulated by the peroxisome-proliferator-activated receptor (PPAR) family of transcription factors. Cancer cells alter metabolic patterns and regulate lipid metabolism through PPARs to promote their own survival and development [58]. PPARγ T166-regulated lipid biosynthesis as an essential pathway for meeting the anabolic demands of the activation and function of macrophages [59]. The mechanism of peroxisomal crosstalk with lipid metabolism regulation via ROS signaling has also been revealed. The β-oxidation of peroxisomes promotes an increase in ROS and the degradation of ATCL by stabilizing PEX2, ultimately affecting lipid droplets and causing them to regulate the homeostasis of fatty acids in cells [60]. In cancers, FAO is usually enhanced in cancer cells along with increased FA synthesis and storage, and FA decomposition and oxidation further provide energy and substrates for cancer cells. Studies have shown that the expression of the FAO-related protein CPT is upregulated in cancer cells and that CPT1A plays an important role in the escape of cancer cells to cytotoxic T cells [61,62].

### 3.1. Fatty Acid Metabolism Reprogramming

In response to the hypoxic tumor microenvironment and a lack of nutrients, cancer cells reprogram FA metabolism and upregulate the de novo synthesis of FA. The accumulation of FAs provides cells with the energy, phospholipids, and lipid mediators required for rapid growth. Studies have found that a variety of key metabolic enzymes involved in fatty acid synthesis and oxidation are upregulated in breast cancer and play an important role in tumor growth, such as SREBP1, ACC1, FASN, SCD, and ELOVL1 [63]. Sterol regulatory element binding proteins (SREBPs) are a family of transcription factors that regulate lipid homeostasis by controlling the expression of a range of enzymes required for the synthesis of endogenous fatty acids, cholesterol, triacylglycerols, and phospholipids. There are three SREBP isoforms, SREBP-1a, SREBP-1c, and SREBP-2, which have different regulatory and activation properties to promote the coordinated regulation of lipid metabolism. The function of SREBP transcription factors is based on the NH (2)-terminally active structural domains (nSREBPs) that are released into the nucleus after cleavage [64]. Among them, SREBP1 mainly regulates adipogenesis-related genes, including ACCs, FASNs, and SCDs [65]. The SREBP1-mediated adaptogenic pathway is significantly activated in HCC, and high SREBP1 protein expression is correlated with a poor prognosis [66]. Our previous study demonstrated that DDX39B directly interacted with and enhanced the stability of the SREBP1 protein by restraining the FBXW7-mediated ubiquitination and degradation of SREBP1 in HCC cells, leading to nuclear translocation and activation of SREBP1 and subsequent de novo lipogenesis [67]. FASN, the rate-limiting enzyme in the fatty acid synthesis pathway, has also been widely reported to promote cancer progression. FASN also plays a key role in maintaining tumor stemness and accelerating tumor growth and invasion in glioblastoma [68]. In many cancer types, including glioblastoma, colorectal cancer, breast cancer, non-small-cell lung cancer, and hepatocellular carcinoma, high levels of ACLY expression are associated with progression [69]. The inhibition of SREBP or restriction of FASN can also trigger the HIF-1α signaling pathway and the unfolded protein response (UPR). In the context of energy deficit-mediated stress, the HIF signaling pathway acts synergistically with AMP-activated protein kinase (AMPK) and mTOR to activate lipid metabolism to rescue lipid-mediated endoplasmic reticulum stress [70]. In addition to SREBPs, other transcription factors are involved in the metabolic regulation of FA. It has been shown that carbohydrate response element binding protein (ChREBP) is activated in cancer cells in response to mitogenic signaling to promote anabolism, including increased lipid biosynthesis [71]. Additionally, MondoA expression induced by the proto-oncogene MYC drives glutamine hydrolysis and lipogenesis in cancer cells to promote tumor growth [72]. Epigenetic mechanisms also play an important role in the regulation of lipid metabolism. The 3K acetylation of ACLY was found to be increased in lung cancer, promoting new lipid synthesis, cell proliferation, and tumor growth. ACLY protein stability is affected by homeostasis between acetylation and ubiquitination [73]. Increased FASN protein levels were associated with decreased FASN acetylation in human HCC samples [74]. ACSL4 was found to be methylated by CARM1 at the R339 site, promoting the ubiquitination of ACSL4. This led to tolerance in tumor cells to iron death therapy [75]. Lipid metabolic reprogramming is regulated by multiple factors.

Lipid metabolic reprogramming provides energy and the precursors required for cancer cell proliferation and biosynthesis, and certain specific fatty acids or lipid molecules may contribute to the maintenance of the stemness characteristics and functions of cancer stem cells (CSCs). The high abundance of unsaturated lipids and fatty acids is a metabolic feature of colon cancer CSCs and ovarian cancer CSCs [76,77]. Fatty acid synthesis also plays a key role in supporting the survival of pancreatic CSCs [78]. Fatty acid oxidation is also important for tumor energy supply, and elevated *β* oxidation in CSCs of HCC both maintains CSCs energy supply and supports CSC self-renewal and drug resistance [79]. Long-chain acyl-coenzyme A synthetases (ACSLs) are localized in mitochondria and endoplasmic reticulum and can catalyze the conversion of FA to Acyl-CoA and further participate in *β*-oxidation and lipid synthesis, playing an important role in lipid metabolism in hepatocytes [80]. The ability of ACSL1 to convert PA to PA-CoA is over-activated under sleep deprivation conditions, further eliciting FAO to promote lung tumor growth and stem cell-like features [81]. Similarly, studies in HCC have found that transcriptional activation of ACSL4 regulates *β*-oxidation of fatty acids and promotes the maintenance of tumor stem cell stemness [82]. Many key enzymes of lipid synthesis and metabolic processes, such as ACLY, FASN, and SCD1, are highly expressed in cancers, promoting cell proliferation while enhancing cell stemness [83,84].

Fatty acid synthesis and utilization occur throughout all stages of tumor development, and the way in which cancer cells utilize lipids is often influenced by complex interactions within the tumor microenvironment and adjacent stroma. In the breast cancer microenvironment, adipocytes, which are highly associated with cancer, interact with cancer cells by releasing a variety of adipocytokines and metabolites and promote tumor development through metabolic remodeling. Co-culturing cancer cells with adipocytes activates lipolysis within the adipocytes, resulting in the release of fatty acids into the extracellular space to support the high demand for lipid metabolism in tumors [85]. Correspondingly, fatty acids secreted into the microenvironment affect cell function and phenotype [86]. Excessive free fatty acids (FFAs) affect cell membrane permeability and fluidity, promoting tissue invasion and metastasis. Studies have shown that FFAs induce lipid accumulation in primary, circulating, and metastatic cancer cells. Furthermore, increased levels of FFAs in plasma are associated with an early elevation of circulating tumor cells and increased lung metastasis of tumors [87]. Monoacylglycerol lipase (MAGL) was found to be highly expressed in invasive human cancer cells and primary tumors, supporting cell membrane synthesis through lipid hydrolysis and modulating signal transduction to promote tumor malignancy [88].

In general, the metabolic reprogramming of fatty acids manifests mainly as an increase in the expression and activity of key enzymes in fatty acid synthesis and metabolic pathways, promoting the de novo synthesis of fatty acids and enhancing lipid catabolism, which further affects the proliferation, movement, and signal transduction of cancer cells.

### 3.2. Cholesterol Metabolism Reprogramming

Cholesterol is an important component of the plasma membrane and plays a key role in maintaining the stability of cell membranes and in biological processes such as signaling. Like other lipids, cholesterol is derived from CoA synthesis within the cell. Intracellular cholesterol is composed of cholesterol synthesized by the cell itself and is ingested in the diet. The liver is the major cholesterol biosynthetic organ, and cholesterol is secreted into the bloodstream as very-low-density lipoproteins (VLDLs), where it is processed into low-density lipoprotein (LDL) and then taken up by peripheral cells via LDL receptors (LDLRs). Excess cholesterol in peripheral tissues can be transported back to the liver as high-density lipoproteins (HDLs) [89]. Liver X receptors (LXRs) are sensors of cholesterol homeostasis. Under normal conditions, when intracellular cholesterol concentrations increase, cells synthesize oxysterols and activate LXR transcription to drive cholesterol efflux and reduce cholesterol influx and synthesis; when oxysterol concentrations decrease, feedback and deactivation lead to the activation of LXRs, allowing them to maintain cholesterol homeostasis. Additionally, in the presence of excess cholesterol, sterol O-acyltransferase 1 (SOAT1) catalyzes the conversion of free cholesterol to cholesterol esters (CEs) and subsequent storage in lipid droplets [90]. Increased CEs and oxysterols are also common phenomena of cancer cells [91]. ACAT1 catalyzes the conversion of excess cholesterol to CEs, promotes tumor growth and metastasis in pancreatic and breast cancer, and is associated with low patient survival [92,93]. Cholesterol esterification due to high SOAT expression effectively promotes the proliferation and migration of cancer cells [94,95]; the inhibition of tumor growth by the inhibition of SREBP-1 via the targeting of SOAT1 has also been demonstrated in GBM [96]. The overexpression of these key enzymes promotes the interconversion of esterified cholesterol and free cholesterol to fulfill the increased demand for cholesterol metabolism in cancer cells.

Studies in melanoma and other cancer types have shown that disturbances in cholesterol balance play an essential role in cancer development. High expression of LDLRs in human breast cancer was found to be associated with reduced recurrence-free survival [97]. Cholesterol uptake is dramatically activated in pancreatic cancer and promotes the malignant progression of pancreatic cancer [98]. Myelin lipidomic analysis of the tumor microenvironment in glioblastomas revealed cholesterol overexpression; in addition, a subpopulation of metabolically reconstituted macrophages with pro-tumorigenic properties was identified, termed lipid-laden macrophages (LLMs). LLMs overload myelin to drive tumor growth via the LXR/ABCA1 lipid transport system [99]. The activation of signals from many oncogenic pathways, such as the RTK/RAS and PI3K/Akt/mTOR pathways, also promotes tumor growth by regulating cholesterol synthesis and can activate SREBP-induced cholesterol biosynthesis [100]. Increased mitochondrial cholesterol is critical for cancer phenotypes, and the PI3K/AKT pathway elevates mitochondrial cholesterol levels to support cancer cell survival through the inhibition of ABCA1-mediated cholesterol efflux [101]. The rate-limiting enzyme of the mevalonate pathway (MVA) for cholesterol biosynthesis, HMGCR, can promote cholesterol biosynthesis. HMGCR expression is upregulated in gastric, glioblastoma, and prostate cancers, and the overexpression of HMGCR promotes cancer cell growth and migration. The effectiveness of HMGCR inhibitors in cancer therapy has been demonstrated [102]. HMGCR is also regulated by SREBP. In addition, the tumor suppressor P53 is involved in the regulation of cholesterol homeostasis. P53 mutants can activate cholesterol synthesis in breast cancer cells through the SREBP2 pathway [103]. SREBP2 not only acts as an important regulator of sterol metabolism but is also subjected to feedback regulation of sterol content. SREBP is synthesized as an inactive precursor that binds to SREBP cleavage activator protein (SCAP) and is retained in the endoplasmic reticulum. When the sterol concentration is reduced, SCAP dissociates from the insulin-induced gene (Insig) and is transferred to the Golgi to be cleaved by S1P/S2P and released into the nucleus to activate lipid synthesis [104]. When cholesterol is too high, cancer cells prevent the accumulation of free cholesterol by inhibiting SREBP2 to reduce HMGCR transcription, and free cholesterol can also block the expression of LDLR. Therefore, cancer cells need to maintain LDLR expression to support proliferation by promoting the uptake of essential FAs. The upregulation of cholesterol metabolism in cancer cells and the microenvironment promotes tumorigenic processes such as tumorigenesis, migration, and angiogenesis [105]. Some cholesterol derivatives also influence tumor development at various stages, with 24-hydroxycholesterol promoting malignant tumor progression by recruiting neutrophils and promoting angiogenesis [106]; 25-hydroxycholesterol promoting the migration and invasion of lung adenocarcinoma cells (LACs) [107]; and 27-hydroxycholesterol linking high cholesterol with LAC metastasis by regulating the NFκB/PPIB axis and the secretion of FGF2 and IL-6 [108]. 25-hydroxycholesterol can promote the killing effect of tumor-specific cytotoxic T lymphocytes (CTLs) and inhibit tumor growth [109]. Importantly, the regulatory mechanism of cholesterol and its derivatives in different situations is not the same.

Lipid rafts are dynamic microdomains rich in lipids and proteins in cell membranes and are composed mainly of cholesterol and sphingomodin, which play important roles in material transport and intracellular signal transduction. Abnormal cholesterol metabolism affects the function of lipid rafts [110]. HCC enhances exogenous cholesterol uptake and promotes endogenous cholesterol synthesis through the LDLR-mediated lipid raft pathway [111]. An increased abundance of lipid rafts in some cancer cells and an imbalance in cellular lipid homeostasis can lead to the disruption of the raft structure and raft-dependent signaling. AKT activation and signaling processes are also dependent on lipid raft regulation. Elevated cellular cholesterol levels lead to increased AKT activation, which could reduce apoptosis in prostate cancer cells [112].

Notably, high-cholesterol diets have been shown to induce steatosis, steatohepatitis, and fibrosis and promote the development of cancer [113]. In summary, sterol regulation of homeostasis is an important aspect of disease and tumor survival. Metabolic reprogramming of cholesterol is characterized mainly by the increased expression of cholesterol-uptake-associated receptors and increased levels of cholesterol synthesis, leading to the abnormal accumulation of metabolites.

### 3.3. Phospholipid Metabolism Reprogramming

Phospholipids are important components of cell membranes and are divided into two major groups: glycerophospholipids (GPLs) and sphingomyelins (SMs). Owing to the demand for membrane lipids caused by abnormal tumor proliferation, phospholipid (PL) synthesis is increased in cancer, using DAG as a precursor. Phospholipid synthesis occurs mainly in the cytoplasmic endoplasmic reticulum. Phosphatidylcholine (PC) and phosphatidylethanolamines (PEs) are the major phospholipids in most cell membranes; together with other phospholipids, such as neutral lipids, these phospholipids form the membrane structure of the phospholipid bilayer. Phospholipid metabolism regulates biological behaviors such as tumor metastasis and drug resistance by modulating changes in membrane lipid composition and the production of bioactive lipid second messengers [114]. The levels of choline cycle metabolites such as choline, phosphocholine, and glyceryl choline are elevated during tumorigenesis, and abnormal choline metabolism has become an important marker of the malignant transformation of tumors. Choline kinase (ChoK) is a metabolic enzyme responsible for the production of phosphocholine (PCho) in eukaryotic cells, and ChoKα activates the first step of PC biosynthesis during phospholipid anabolism. ChoKα has been observed to be activated in various cancers, and the overexpression of ChoKα contributes to tumor progression, metastasis, and invasion [115]. Growth factor receptor-driven cancer relies on LPCAT1 for plasma membrane synthesis and tumor signaling by increasing the content of saturated phosphatidylcholine [116].

Proliferating cancer cells are exposed to relatively high levels of phosphatidylserine (PS) on the ectoplasmic surface of the plasma membrane [117]. PS on the inner leaflet of the membrane plays a crucial role in the activation of key kinases, such as PKC, PDK1, and AKT, and is also an interacting molecule for various signaling proteins [118]. Multi-omics data revealed that the most significant changes in ovarian cancer cells co-cultured with adipocytes were GPLs, which promote metastasis by generating glycerol-3-phosphate (G3P), a precursor of the membrane and signaling components in fat-rich tumor microenvironments. Knockdown of G3P acyltransferase 3 has been shown to inhibit metastasis in an ovarian cancer xenograft model [119]. Phospholipid metabolism also affects immune cell function. PD-1 signaling was found to lead to impaired phospholipid metabolism and increased ferroptosis in CD8^+^ T cells through the inhibition of phospholipid phosphatase 1 (PLPP1), which in turn affects the antitumor function of CD8^+^ T cells [120]. In the metastatic process of cancer cells, the fusion of cancer cells with neighboring cells can help them to better metastasize and spread, and the heterozygosity of cancer cells with immune cells can allow them to escape recognition and clearance by the immune system [121]. This process relies on the fluidity of the cell membrane. ACSL4 is specifically elevated in TNBC cells, and ACSL4 promotes the preferential incorporation of polyunsaturated fatty acids into phospholipids, thereby altering the composition and properties of membrane phospholipids. This remodeling of phospholipid metabolism results in increased cell membrane phospholipid unsaturation and enhanced membrane fluidity. The localization and interaction of relevant membrane proteins in lipid raft structures promote cancer cell invasion and metastasis [122].

The metabolic reprogramming of phospholipids mainly manifests as changes in enzyme activities in the phospholipid metabolism pathway, leading to changes in the composition and content of different phospholipids. This reprogramming not only contributes to cancer cell growth and division but also may affect cancer cell invasion, metastatic capacity, and tolerance to treatment. Lipid metabolic reprogramming may provide a specific lipid environment for tumor stem cells. For example, specific lipids may be involved in regulating stemness-related signaling pathways, enhancing the self-renewal and anti-apoptotic capacity of stem cells, or attenuating stemness. The level of PUFA in ovarian cancer CSCs was significantly greater than that in non-CSCs, and inhibition of the signal transduction pathway of NF-κB-regulated lipid desaturase could effectively kill CSCs and block their ability to initiate tumors [77]. In turn, tumor stemness further promotes lipid metabolic reprogramming. The unique properties of stem cells enable them to adapt better to metabolic stress and drive changes in lipid metabolism in a direction that is more favorable for their survival and development. They may enhance the activity of key enzymes, such as fatty acid synthase, to promote lipid synthesis and accumulation by regulating the expression of relevant genes.

Lipid metabolic reprogramming may be conditionally involved in the development of tumors at different stages. Here, we preliminarily summarize the roles of lipid metabolic enzymes in tumors in Table 1. In conclusion, the core of lipid metabolic reprogramming lies in its adaptability. It enables organisms to rapidly adjust the lipid metabolic pattern under different metabolic stressors to ensure the survival and function of cells and tissues. Through this reprogramming, organisms can better cope with various unfavorable situations, such as nutritional deficiencies and energy shortages, and enhance their ability to survive.

## 4. Association of Oxidative Stress and Lipid Metabolism

Lipid metabolism homeostasis interacts closely with redox homeostasis. On the one hand, upregulated lipid metabolism enzymes in tumors can over-catabolize ROS, and activated lipid synthesis can generate ROS to a certain extent via FAO; meanwhile, excessive ROS can further contribute to lipid peroxidation (LPO). On the other hand, the ROS generated by moderate oxidative stress can also act as signaling molecules to activate a series of stress-responsive pathways to promote the survival and adaptation of cancer cells to adverse environments. Proteins and lipids are the main targets of oxidative stress attacks, and the accumulation of protein and lipid metabolites via oxidative modification eventually leads to abnormal signaling pathways and further induces cell or organismal damage. Under oxidative stress, phospholipids of polyunsaturated fatty acids (PUFA-PLs) are easily oxidized through free radical-induced lipid peroxidation to form oxidation products. This results in the accumulation of lipid peroxides in the cells. The excessive accumulation of peroxides increases cell membrane instability and ultimately results in cell death; this is a mode of cell death in which oxidative stress and lipid oxidation crosstalk is known as ferroptosis. The concept of ferroptosis was first introduced in 2012; it is defined as a mode of programmed cell death characterized by the iron-catalyzed over-oxidation of PUFA-PLs [123]. Oxidative stress is involved not only in ferroptosis but also in traditional cell death types, such as apoptosis. The mode of cell death depends on multiple factors. Because ferroptosis is a form of programmed cell death that depends on iron and lipid peroxidation, cells are prone to ferroptosis when the intracellular iron homeostasis is unbalanced, the activity of the antioxidant system is dysregulated (especially GPX4 and SLC7A11), and lipid metabolism is dysregulated. Under oxidative stress, ROS signaling induces apoptosis by activating BCL-2, BAX, P53, and other genes involved in apoptosis-related pathways. Sometimes, ferroptosis and apoptosis are not completely independent, and the cell may exist in multiple states that jointly determine cell fate.

PL is a fundamental component of the cell membrane. If phospholipid peroxides (PLOOHs) cannot be effectively neutralized, their accumulation destroys the integrity of the plasma membrane, leading to cellular ferroptosis. Here, the oxidation of PL occurs on PUFA-acyl chains and forms PUFA-PLs. PUFA-PLs can be converted to PLOOHs through enzymatic and non-enzymatic lipid peroxidation reactions; all mammalian cells contain some level of PUFA-PLs and bioactive iron [124]. It is evident that iron metabolism and lipid metabolism are extremely important parts of mammalian biological processes. This unique pattern of cell death is driven by iron-dependent phospholipid peroxidation and is regulated by a variety of cellular metabolic pathways, including redox homeostasis, iron metabolism, mitochondrial activity, and the metabolism of amino acids, lipids and glucose, as well as by a variety of disease-related signaling pathways [125]. The synthesis and peroxidation of PUFA-PLs, iron metabolism, and mitochondrial metabolism are prerequisites for driving ferroptosis [126]. During ferroptosis, the peroxidation of PUFA-PLs and the production of 4-HNE or MDA lead to cell membrane instability and permeability, leading to cell death. The glutathione peroxidases family contains eight members (GPX1-GPX8) in mammals, which interrupts the free radical chain reaction and inhibits lipid peroxidation, usually using GSH as a reductant. In particular, GPX4 functions by protecting cell membranes from oxidative damage and has the unique ability to convert lipid peroxides into lipid alcohols [127]. In addition, the intracellular cystine/glutamate reverse transporter system (System xc-) also plays a crucial role in maintaining redox homeostasis [40]. This system consists of two proteins—the light-chain subunit SLC7A11 (xCT) and the heavy-chain subunit SLC3A2 (4F2)—where the SLC7A11 protein is responsible for transport activity, importing cystine, and exporting glutamate for glutathione biosynthesis at a 1:1 ratio [128]. Blocking these important intracellular redox pathways effectively induces ferroptosis. Moreover, lipid peroxidation depends on the balance between the rate of formation of lipid oxidation products and the rate of detoxification by enzymes such as GSH-Px, GST, or AKR [129].

Intracellular lipid peroxidation consists of two components: non-enzymatic peroxidation of lipids and enzyme-catalyzed lipid peroxidation. The former is the peroxidation of PUFAs initiated by ROS. Centered around the Fenton reaction and the Harber-Weiss reaction, ^•^OH produced by the reaction of H_2_O_2_ and Fe^2+^ extracts hydrogen from PUFAs to form a carbon-centered phospholipid radical (PL^−^). PL^−^ reacts with O_2_ to form the phospholipid peroxyl radical (PLOO^−^). PLOO^-^ separates hydrogen from another PUFA to form a phospholipid peroxyl (PLOOH) and a new PL^−^, which can again react with O_2_. In the process of ferroptosis, an imbalance in the locking reaction eventually leads to the breakage of the cell membrane. In the process of enzyme-catalyzed lipid peroxidation, free PUFAs can synthesize different kinds of oxidized lipids through mono-oxygen or dual-oxygen under the action of three enzymes: cyclooxygenase (COX), lipoxygenase (LOX), and cytochrome P450 (CYP) [130]. For example, LOX catalyzes the peroxidation of PUFAs to generate PLOOHs. In the presence of Fe^2+^, PLOOH can be decomposed into an alkoxyl phospholipid radical (PLO^-^), which can then attack nearby PUFAs in a chain reaction to promote lipid peroxidation [131]. The accumulation of lipid peroxides is an important manifestation of lipid metabolism disorders. Studies have shown that oxidative stress and electrophilic lipid peroxidation products, such as HNE, also play important roles in inducing cell cycle arrest, differentiation, and apoptosis in cancer cells [132].

The effect of lipid peroxidation is also twofold: on the one hand, it can cause damage to cells and tissues, such as destroying the integrity of the cell membrane, leading to cell dysfunction and even death, and can play a negative role in the occurrence and development of many diseases. On the other hand, the accumulation of lipid peroxides and the production of ROS act as a signal to induce an adaptive response in cells to cope with oxidative stress and to regulate lipid homeostasis in a feedback mechanism.

### 4.1. Oxidative Stress Affects Lipid Metabolism

Oxidative stress and dysregulated lipid metabolism together drive the development of a wide range of diseases. Mitochondrial homeostasis links redox reactions in tandem with lipid metabolism. Mitochondrial function is critical for the development of lipid peroxidation and oxidative stress [133]. Mitochondria also host many other organisms and many other important metabolic processes, such as the electron transport chain, the TCA cycle, and glutamine metabolism [34,134]. Glutamine is a key component of SREBP, and it is required for SREBP activation and adipogenesis [135]. The primary site where this important biological process of FAO occurs is also the mitochondria. Most oxysterols are produced from cholesterol by autoxidation or specific microsomal or mitochondrial oxidation, which usually involves cytochrome P-450 species [136]. Phospholipid transport between mitochondria and other organelles is an essential process in biosynthesis, and the field of phospholipid synthesis is usually restricted to the ER. For example, PS is synthesized in the region of the mitochondrial-associated membrane (MAM) and decarboxylated to PE in mitochondria; this process relies on protein complexes linking the two organelles to facilitate lipid transfer [137]. Mitochondria may drive oxidative stress and lipid peroxidation by participating in a variety of biological processes. Hypoxia, oxidative stress, and inflammatory conditions all contribute to increases in ROS and oxidative stress. This oxidative-stress-induced metabolic remodeling may show different adaptations in different stages or situations.

#### 4.1.1. Effects on Lipid Uptake and Synthesis

Under oxidative stress conditions, the cellular damage caused by lipid peroxides formed by abnormal lipid metabolism and their accumulation may activate certain lipid compensatory mechanisms and de novo lipids to maintain normal cell function. Increased uptake of FA and LD accumulation was observed when CSCs were exposed to a hypoxic environment. HIF-1α and pyruvate dehydrogenase (PDH) block the entry of pyruvate into the tricarboxylic acid cycle, thereby preventing glucose-induced FA synthesis. However, cells grown under a hypoxic environment relied almost exclusively on the reductive carboxylation of glutamine-derived α-ketoglutarate for de novo lipogenesis; acetyl-CoA synthetase 2 catalyzes the conversion of intracytoplasmic acetic acid to acetyl-CoA, which also contributes to FA synthesis. These results identify a critical role for oxygen in regulating carbon use to produce AcCoA and support lipid synthesis in mammalian cells [138]. Heterozygous mutations in NADP-dependent isocitrate dehydrogenases (IDHs) are characteristic of the majority of diffuse gliomas, and numerous metrics have demonstrated that IDHm gliomas are in a state of oxidative stress, with significant changes in phospholipid metabolites accompanying the histological data [139]. NRF2, an important reactive element in oxidative stress, is also involved in the regulation of lipid metabolism. Oxidative stress was found to promote Nrf2 recruitment in the SREBP1 promoter in adipose tissue from mice with high-fat diet-induced or genetically (ob/ob)-induced obesity, as well as in obesity patients, inducing transcription of target genes and subsequent adipogenesis [140]. Nrf2 modulates metabolic reprogramming of HepG2 cells by regulating SREBP1 through PI3K-AKT-mTOR signaling [141].The oxidative stress and lipid metabolic stability in cancers may be an entry point for therapy.

#### 4.1.2. Effects on Lipid Metabolism and Catabolism

HIF-mediated signal transduction upregulates the expression of hypoxia-inducible protein 2 (HIG2), leading to the expression of adipose triglyceride lipase (ATGL)-mediated lipolysis and the induction of LDs accumulation and absorption. In addition, ATGL reprograms β-oxidation and de novo biosynthesis of FAs [142]. HIG2-mediated inhibition of ATGL reduces LD degradation and mitochondrial oxidation of FAs, enabling hypoxic cancer cell survival by reducing ROS production. Decreased ATGL expression in non-small-cell lung cancer, pancreatic cancer, and other cancers is associated with cancer development [143]. Cholesterol oxidase (CHO)-loaded Co-PN3 monatomic nanoenzyme (Co-PN3SA/CHO) catalyzes O_2_ and H_2_O_2_ to produce ROS through their oxidase-like and Fenton-like properties. The O_2_ produced by this catalytic enzyme can combine with excess cholesterol and produce more H_2_O_2_ and ·OH under the catalysis of CHO, thereby enhancing oxidative damage to cancer cells. The depletion of cholesterol in cancer cells is caused by the cascade reaction; it can also destroy the integrity of lipid rafts and inhibit the proliferation and metastasis of cancer cells [144].

#### 4.1.3. Effects on Lipid Transport and Storage

The remodeling of cholesterol metabolism that occurs under conditions of oxidative stress also protects cells from oxidative stress. In ALK^+^ anaplastic large cell lymphoma (ALCL) cells and primary tumors, squalene accumulation induced by the inhibition of the cholesterol biosynthesis pathway enzyme squalene cyclooxygenase (SQLE) alters the cellular lipid distribution and protects membrane lipids from chemical modification or remodeling, avoiding ferroptosis triggered by the over-oxidation of membrane PUFAs and providing a growth advantage under oxidative stress conditions and in tumor xenografts [145]. ECI2 is a protein present in mitochondria and peroxisomes, and studies have demonstrated its involvement in peroxisome-driven ether lipids in ferroptosis in cancer. In colorectal cancer, the ECI2 protein was found to promote CoA re-entry into the *β*-oxidation cycle and participate in peroxisomal lipid metabolism [146]. In addition to storing energy, lipid droplets play important roles in alleviating cellular stresses, such as lipotoxic stress, ER stress, and starvation. Therefore, dysfunction or excessive accumulation of lipid droplets is associated with many metabolic diseases and cancers [147]. Lipopolysaccharide-binding protein (LBP) mediates the selective isolation of long-chain polyunsaturated fatty acid-triglyceride (UFA-TG) in LBP into LDs, which effectively prevents lipolysis under oxidative stress. LBP acts as an antioxidant to control lipid homeostasis and protects against oxidative stress by coupling to redox signaling and lipid metabolism [148].

**Figure 2:**
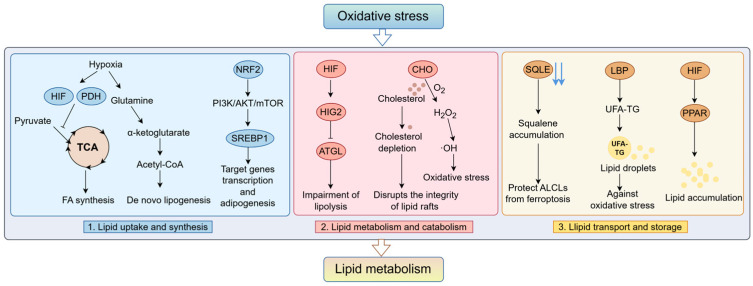
Oxidative stress affects lipid metabolism. Oxidative stress can regulate the activities of related enzymes through a variety of signaling pathways, resulting in lipid metabolism disorders, and further affect the proliferation, migration, and invasion of tumors through lipid metabolism reprogramming.

In summary, the influence of oxidative stress can regulate the activity of related enzymes through a variety of signaling pathways, leading to lipid metabolism disorders (Figure 2). Oxidative stress is often associated with mitochondrial damage because mitochondria are the primary sites of redox generation in the electron transport chain. Mitochondrial dysfunction may lead to disordered lipid metabolism, which in turn leads to the abnormal accumulation of lipids in cells. Oxidative stress, mitochondrial dysfunction, and the HIF1α–PPARγ pathway have been shown to be key mediators of iron-induced abnormal lipid accumulation [149].

### 4.2. Abnormal Lipid Metabolism Triggers Oxidative Stress

**Figure 3:**
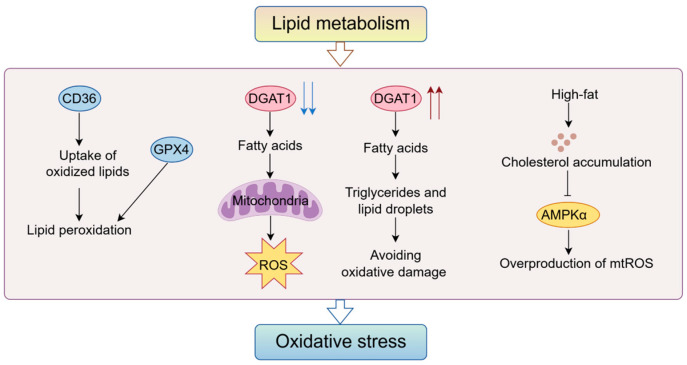
Abnormal lipid metabolism triggers oxidative stress. Lipid oxidation and lipid metabolism disorders can lead to increased production of free radicals, which can trigger oxidative stress.

Lipid oxidation and lipid metabolism disorders can lead to increased production of free radicals, which can trigger oxidative stress (Figure 3). The accumulation of lipid peroxides in the cellular environment that cannot be removed can lead to cell death. Conversely, lipid metabolism alleviates oxidative stress by either increasing storage or decreasing synthesis. Owing to elevated levels of lipid oxidation in tumors, the uptake of oxidized lipids by CD8^+^ TILs via CD36 is increased, leading to lipid peroxidation. Blocking CD36 or GPX4 overexpression inhibits lipid peroxidation, enhances the effector function of CD8^+^ TILs, and strengthens the anti-tumor effect [150]. The final critical step in TG synthesis is regulated by two diacylglycerol acyltransferases, DGAT1 and DGAT2. Studies have shown that glioblastoma (GBM) up-regulates DGAT1, which stores excess fatty acids as triglycerides and lipid droplets. Inhibition of DGAT1 disrupts lipid homeostasis and leads to excess fatty acids entering mitochondria for oxidation, resulting in ROS generation, mitochondrial damage, and apoptosis. Upregulation of DGAT1 protects the GBM from oxidative damage and maintains lipid balance by promoting the storage of excess fatty acids [151]. A high cholesterol diet leads to increased oxidative DNA damage in the liver [152]. Cholesterol accumulation due to a high-fat diet inhibits AMPKα activity in macrophages, leading to overproduction of mtROS, which in turn activates NLRP3 inflammatory vesicles, aggravates the inflammatory response and increases tumor burden [153]. Cholesterol-induced oxidative stress and the promotion of cellular dysfunction may contribute to cancer induction.

### 4.3. New Strategies for Cancer Therapy Targeting Lipid Metabolism

Therapeutic options targeting key enzymes of the lipid metabolic pathway are highly important, and the field is still being explored. Acetyl-CoA carboxylases (ACCs) produce malonyl-CoA, an intermediate metabolite that is both a substrate for fatty acid synthesis and a negative regulator of fatty acid oxidation. Inhibition of ACC selectively induces growth arrest and cytotoxicity in prostate cancer cells, but this does not happen for premalignant and nonmalignant cells [154]. HMGCR is the rate-limiting enzyme of cholesterol synthesis, and its overexpression promotes the growth and migration of cancer cells. Statins are competitive inhibitors of HMGCR and have been shown to be effective in inhibiting tumor growth [102]. In addition, cancer cells have higher levels of redox and lipid metabolism than normal cells because of their imbalanced proliferative demands and the close association of ferroptosis with redox and lipid metabolism. Homeostasis is the source of its feasibility for use in cancer therapy. Intervention in the metabolic mechanisms that protect cells from feasibility has emerged as a promising therapeutic tool in preclinical studies. The feasibility inducers erastin, RSL3, and their analogs promote cancer cell death in a variety of cancers, and many drugs such as sorafenib and dexamethasone have been shown to have therapeutic effects [155]. Interestingly, the inhibition of LD biogenesis by ferroptosis-inducing RSL3 agents may be a promising approach for the treatment of TNBC [156]. FASN inhibits sorafenib-induced ferroptosis by binding to HIF1α and promoting HIF1α nuclear translocation to enhance SLC7A11 transcription, whereas the FASN inhibitor orlistat has a significant synergistic antitumor effect with sorafenib and reverses sorafenib resistance [157]. Moreover, our recent study revealed that DDX39B protected against sorafenib-induced ferroptosis through enhancement of antioxidant capacity and maintenance of mitochondrial function, and this function of DDX39B was dependent on its ATPase activity to facilitate the splicing and cytoplasmic export of GPX4 mRNA in HCC [158].

An increasing number of therapeutic strategies based on lipid metabolism are emerging (Table 2). Metabolic homeostasis in cancer cells is heterogeneous, and the degree of dependence of different cancer cells on different types of metabolism is extremely promising and challenging in research on tumor-specific therapies.

#### 4.3.1. Targeted Therapy Based on Lipid-Metabolizing Enzymes

Although cancers vary greatly in type and etiology, cancer cells are often characterized by metabolic abnormalities. In particular, there are changes in lipid metabolism. The onset and progression of cancer are often accompanied by significant changes in the expression and activity of enzymes involved in the cellular fatty acid balance. There are many enzymes and substrates involved in the process of lipid metabolism, which are potential intervention points for clinical research.

Several drugs have been used to intervene in lipid metabolism. The main lipid-lowering drugs are statins, cholesterol uptake inhibitors, fibrates, antioxidants, niacin, and PCSK9 inhibitors. The inhibitory effect of the statin lovastatin on the DNA repair process is due to blockage of the mevalonate pathway. A subcutaneous xenograft mouse model of gallbladder cancer showed that lovastatin-induced HMGCR ablation inhibited tumor growth in vivo and promoted the efficacy of cisplatin, significantly prolonging the survival time of the mice [152]. Simvastatin inhibits the activity of hydroxymethylglutaryl coenzyme A (HMG CoA) reductase and prevents cholesterol synthesis. Simvastatin can inhibit the proliferation of HCC cells by inhibiting PKM2-mediated glycolysis, inhibiting the HIF-1α/PPAR-γ/PKM2 axis, and increasing the sensitivity of HCC cells to sorafenib [159]. A reduction in cholesterol content in adrenocortical carcinoma (ACC) by simvastatin can prevent the production of estradiol, inhibit mitochondrial function, and induce cell apoptosis [160]. Akt protein kinase is critical for the pro-growth and anti-apoptotic effects of leptin in esophageal cancer cells, and atorvastatin inhibits esophageal carcinogenesis by reducing the leptin-induced activation of Akt [161]. Fatostatin, a specific inhibitor of SREBPs, inhibits SREBP maturation by blocking the translocation of SCAP from the endoplasmic reticulum to the Golgi. Fatostatin enhances the sensitivity of endometrial cancer to luteinizing hormone and reverses luteinizing hormone resistance by inhibiting SREBP1 [162]. Fatostatin can also induce ferroptosis by inhibiting the AKT/mTORC1 signaling pathway to inhibit GPX4 synthesis in glioblastoma [163]. Inclisiran is the first siRNA drug approved for the treatment of chronic diseases in the world, and it is also the only siRNA drug approved in China thus far [164]. Inclisiran specifically blocks the transcription of PCSK9 mRNA and thus inhibits PCSK9 synthesis. Currently marketed PCSK9 inhibitors include alirocumab, evolocumab, and inclisiran, as well as small-molecule nucleic acid drugs and vaccines in development [165]. Preclinical studies have demonstrated that PCSK9 can modulate immune responses by interacting with immune cells and TME components and have shown that there are synergies among alirocumab, evolocumab, and existing immunotherapies [166]. A retrospective cohort study revealed that PCSK9 inhibitors, particularly alirocumab and evolocumab, reduce the risk of melanoma skin cancer (NMSC) [167]. Researchers have developed CaCO_3_-based nanoparticles (DECP) coated with the immunogenic cell death (ICD) inducers DOX and evolocumab, which can inhibit hepatocellular carcinoma growth. Peritumoral administration of DECP in combination with intravenous αPD-L1 therapy enhances the immune response to distant tumors and has shown antitumor effects [168]. Results based on Clinical Practice Research Datalink analyses suggest that cholesterol uptake inhibitors may be associated with a reduced risk of hepatocellular carcinoma. Animal studies have also shown that the cholesterol uptake inhibitor ezetimibe inhibits hepatic tumor growth and angiogenesis associated with hypercholesterolemia [169]. TVB-264, a FASN inhibitor, has entered phase II trials in glioblastoma and can be safely and effectively combined with bevacizumab [170].

FAO inhibitors can be used as effective therapeutic agents for treating hematological malignancies. Studies have shown that the reduction in fatty acid oxidation by etomoxir or ranolazine can inhibit the proliferation of human leukemia cells and sensitize human leukemia cells to inducers of apoptosis in vitro. The use of fatty acid synthesis/lipolysis inhibitors orlistat increases the sensitivity of leukemia cells to apoptosis inducers [171]. On the basis of the important role of lipid droplets in promoting tumor metastasis, miR-532-5p inhibits the epithelial–mesenchymal transition and lymphangiogenesis by regulating LD accumulation in cervical cancer (CC) patients with lymph node metastasis (LNM). Combined treatment with miR-532-5p and the FASN inhibitor orlistat can further suppress tumor growth and LNM [172,173]. The efficacy of novel drug combination therapy with LXRβ agonist and anti-apoptotic Bcl-2 inhibitor has also been confirmed in the treatment of malignant solid tumors [174]. The fatty acid binding protein-4 (FABP4) inhibitor can inhibit TME lipid transport and reduce tumor regeneration. By further targeting SCD1 in vivo and promoting oxidative-stress-induced ferroptosis, tumor regeneration can be completely eliminated [175]. Interestingly, not only the inhibition but also the upregulation of genes involved in the regulation of lipid metabolism may lead to therapeutic sensitization. Studies have shown that rosiglitazone-induced radiosensitivity in pancreatic cancer is achieved by increasing the expression and nuclear translocation of PPARγ, which is related to the abnormal lipid metabolism caused by the PPARγ-induced upregulation of FABP4 [176]. Another study showed that pioglitazone induced PD-L1 autophagic degradation in a PPARγ-dependent manner, resulting in increased colorectal tumor immunotherapy [177]. SREBP1 inhibitor Betulin can inhibit the proliferation and metastasis of pancreatic ductal adenocarcinoma (PDAC) cells and attenuate the process of liver metastasis in vivo [178]. Betulin inhibits lung metastasis by inducing cell cycle arrest, autophagy, and apoptosis in metastatic colorectal cancer cells [179]. Daralutamide promotes ferritin deposition and inhibits the transcription of FASN by downregulating SREBP1, mediates phospholipid remodeling through fatty acid homeostasis, and then induces ferroptosis. In the meantime, the synergistic antitumor effect of combination therapy with darolutamide and FINs was confirmed in PCa organoids and a mouse xenograft model [180].

Lipid metabolism is highly heterogeneous, and genetic polymorphisms in key enzymes of lipid metabolic pathways may exist in different individuals; therefore, personalized medicine is increasingly important in targeting key enzymes of lipid metabolic pathways. In addition, combination therapy is also an important direction. Combining drugs targeting key enzymes involved in lipid metabolism with other therapeutic means may produce synergistic effects. Oxidative stress is a common feature of many diseases, so antioxidants are also a promising therapeutic strategy. Current antioxidant drugs are broadly classified into three categories: antioxidants that directly scavenge reactive oxygen species, inhibitors of ROS synthesis, and ROS metabolism analogues. Regulating the cellular redox state by targeting ROS and their regulatory mechanisms is considered a promising strategy for cancer therapy [181]. Cellular oxidative damage can be monitored by detecting the photoemission of ^1^O_2_, the end-product content of lipid peroxidation, and the release of glutathione disulfide. These assays help us understand redox homeostasis in intact cells or organs and provide a better basis for designing targeted clinical strategies. The combination of lipid metabolism-related drugs and antioxidants may reduce the adverse effects of oxidative stress on lipid metabolism or amplify the killing effects of the drugs. The realization of precision medicine requires continuous exploration and utilization of cutting-edge advances to provide new ideas and methods for improving the treatment effect of related diseases.

#### 4.3.2. Redox Homeostasis Intervenes in Lipid Metabolism

Atorvastatin (ATO) has been used for sustainable ROS production to enhance the antitumor efficacy of PD-L1 silencing. They reported that Ato activated AMPK and promoted mitochondrial fatty acid oxidation (FAO) to produce ROS, which was previously highly inhibited in cancer cells. The ROS from FAO further activates AMPK and establishes a positive feedback mechanism for sustainable ROS production [182]. ATO could be able to promote apoptosis by inhibiting the antioxidant Nrf2 pathway, which induces ROS accumulation and mitochondrial dysfunction in HepG2 cells [183]. CPI-613 showed anticancer activity in pancreatic cancer cells by activating AMPK signaling and triggering ROS-related apoptosis while increasing autophagy and inhibiting ACC-regulated lipid metabolism [184]. IR induces not only ROS but also the expression of ACSL4, a lipid metabolism enzyme required for iron-dependent lipid peroxidation, leading to lipid peroxidation. As an adaptive response, IR also induces the expression of the ferroptosis inhibitors SLC7A11 and GPX4 [185]. Some genes show different adaptations in response to changes in environmental oxygen. Mitochondrial oxidative phosphorylation (OXPHOS) complex I is required for cell survival at high oxygen pressures, and lipid metabolism and metabolic pathways responsible for ether lipid synthesis in the peroxisome are selectively required for cellular adaptation in hypoxic environments [186]. Lipid metabolic homeostasis is influenced by redox homeostasis, which in turn results in adaptive metabolic reprogramming in different environments. Owing to the large variety of small molecules involved in lipid metabolism and redox systems and their widely varying chemical properties—and in the hopes of addressing the problem in which small molecules still lack accurate and broad-spectrum detection methods—a team established a method for the detection of a wide range of redox-associated small molecules by using an LC–MS-based targeted metabolomics strategy. The method achieves the high-throughput analysis of 22 small molecule compounds, including redox pairs, RNS-related molecules, lipid peroxides, etc., and can be used for the detection of various types of biological samples, such as cells, animal tissues, and body fluids [187]. All these studies will advance the field of precision medicine targeting lipid metabolism. For example, small molecule inhibitors or agonists are used to regulate the activity of key enzymes to achieve balanced control of lipid synthesis and catabolism.

**Table 2:** Targeted therapy based on lipid-metabolizing enzymes.

Target	Drug	Effect	Study Phase	Condition	
Intervention in lipid synthesis	TVB-2640	FASN inhibitors	Phase II	Glioblastoma	[170]
	Lovastatin	Inhibition of cholesterol synthesis	Preclinical	Gallbladder	[152]
	Simvastatin	Promotes apoptosis	Preclinical	Melanoma, hepatocellular carcinoma, adrenocortical carcinoma	[159,160,188]
	Orlistat	FSAN inhibitor	Preclinical	Hematologic tumor, lung cancer	[171,172,173]
	Fatostatin	SREBP1 inhibitor	Preclinical	Endometrial cancer, glioblastoma	[162,163]
	Betulin	SREBP1 inhibitor	Preclinical	Adenocarcinoma, colorectal cancer	[178,179]
	Rosiglitazone	PPARγ agonist	Preclinical	Pancreatic cancer	[176]
	Pioglitazone	PPARγ agonist	Preclinical	Colorectal cancer	[177]
	Darolutamide	SREBP1 inhibitor	Preclinical	Prostate cancer	[180]
	CPI-613	ACC inhibitor	Preclinical	Pancreatic cancer	[184]
Intervention in lipid metabolism	Ezetimibe	Enhanced immune cell infiltration for immunotherapy	Preclinical	Hepatocellular carcinoma, colorectal cancer	[166,168]
	Alirocumab	Enhanced immune cell infiltration for immunotherapy	Preclinical	Colorectal cancer	[166]
	Atorvastati	Inhibition of lipid acylation affects signaling pathways	Preclinical	Esophageal cancer	[161]
	Etomoxir	Inhibition of FAO	Preclinical	Hematologic tumor	[171]
	Ranolazine	Inhibition of FAO	Preclinical	Hematologic tumor	[171]
	Ezetimib	Inhibition of tumor growth and angiogenesis associated with hypercholesterolemia	Preclinical	Hepatocellular carcinoma	[169]
	BD62694	FABP4 inhibitor	Preclinical	Glioblastoma	[175]

#### 4.3.3. Dietary Intervention in Lipid Metabolism

As research into lipid metabolism in cancer continues to intensify, studies relating diet to disease and cancer are being revealed. Dietary interventions may, in turn, affect tumor development through lipid metabolism. Exposure to a high-fat diet alters normal liver metabolism, and this metabolic milieu, similar to that found in hepatocellular carcinoma (HCC), may provide conditions for carcinogenesis and tumor development in liver tissue [189]. A high-cholesterol diet induces NASH and exacerbates hepatocellular carcinoma development via calcium signaling [190]. A number of cancers are highly correlated with diet and obesity, including colorectal, endometrial, breast, and pancreatic cancers [191,192,193].

However, some studies have demonstrated that certain sources of healthy lipids can improve overall metabolic status, thereby reducing the risk of tumorigenesis or slowing its progression. Since other membrane lipids such as phosphatidylcholine and phosphatidylethanolamine may affect the immune escape of tumors, altering the composition of membrane sphingolipids through pharmacological or dietary interventions may be a viable strategy to improve the response to immunotherapy [194]. For example, increasing the intake of the polyunsaturated fatty acids ω-3 and ω-6 and decreasing the intake of saturated and trans fatty acids can improve fatty acid composition and reduce inflammatory reactions and oxidative stress [195]. ω-3 has been shown to inhibit the proliferation of lung cancer cells and reduce the toxicity of chemotherapy [196]. Lipid levels in the plasma and tumors of mice decrease after caloric restriction (CR), and CR causes an imbalance between unsaturated and saturated fatty acids by compromising the activity of tumor SCD, thereby slowing tumor growth [197]. The Women’s Health Initiative (WHI) Dietary Modification (DM) clinical trial revealed that a low-fat dietary pattern, with increased intake of vegetables, fruits, and grains, reduces the risk of death from breast cancer in postmenopausal women [198]. A prospective cohort study with a mean follow-up time of 8.8 years investigated the association of adherence to a low-fat diet (LFD) and the intake of different fat components with the incidence of lung cancer and its subtypes in adults aged 55 years and older. Studies have shown that adherence to a low-fat diet can reduce the risk of lung cancer, especially for smokers. In middle-aged and older Americans, highly saturated fatty acid intake may increase the risk of lung cancer, especially SCLC [199].

Clarifying the relationship between dietary intervention and lipid metabolism changes and tumor development not only helps us to better understand the mechanism of tumor occurrence and development but also opens up broad prospects for the development of new antitumor therapies based on lipid metabolism regulation. Such research will provide an important basis for formulating more targeted prevention and treatment strategies.

## 5. Conclusions and Future Directions

The reprogramming of lipid metabolism in cancer cells is extremely closely related to redox homeostasis. Increased metabolic activity is accompanied by ROS production, which in turn affects lipid metabolic homeostasis at multiple levels. Oxidative stress refers to the excessive production of highly reactive molecules such as ROS and RNS in the body when the body is subjected to various harmful stimuli, resulting in an imbalance between the oxidative and antioxidant systems. Lipid peroxidation is an important aspect of oxidative stress.

In conclusion, the causes of oxidative stress in cancers mainly include the following aspects. First, the metabolism of cancer cells is abnormally active, especially the enhancement of glycolysis, which leads to the production of large amounts of ROS. This high metabolic state increases electron leakage in the electron transport chain, which in turn triggers oxidative stress. Cancer cells face a hypoxic environment when rapidly proliferating, and the activation of hypoxia-inducible factors and other relevant pathways can induce the production of ROS. Inflammatory reactions in the tumor microenvironment can also promote the release of a variety of inflammatory factors and ROS in immune cells to exacerbate oxidative stress. In addition, mitochondrial dysfunction is a relatively common phenomenon in cancer cells. Mitochondria may become dysfunctional in the process of energy generation to produce excessive ROS. Genetic changes in cancer cells, such as the activation of oncogenes or the inactivation of oncogenes, may directly or indirectly affect the intracellular redox balance, leading to the occurrence of oxidative stress. In addition, external environmental factors, such as radiation and chemical substances, may also cause elevated levels of intracellular oxidative stress while inducing tumor formation. In summary, multiple factors interact and together contribute to the persistence of oxidative stress in tumors.

The reprogramming of lipid metabolism is also dependent on the functional stability of related organelles within the cell. Not only does mitochondrial homeostasis link redox reactions to lipid metabolism, but interactions between lipid metabolism-related organelles such as the ER, lipid droplets, lysosomes, and peroxisomes, as well as the key enzymes involved in lipid regulation in each of these organelles—also play important roles. In summary, the maintenance of redox homeostasis through mitochondrial function to promote lipid metabolism regulation, the regulation of lipid metabolism by peroxisomes, and the storage of fatty acids in lipid droplets to prevent intracellular peroxidative damage have all been shown to be effective strategies in avoiding oxidative stress.

Lipids have important biological functions in vivo, such as providing energy storage, transmitting signals, and supporting membrane synthesis and structural functions. Excessive lipids can promote the occurrence and development of tumors and affect normal cell function through lipotoxicity. In the process of cell biology, tumors will face different metabolic pressures. Lipid reprogramming promotes lipid catabolism and anabolism to achieve energy and oxidative stress protection. Cancer cells also use lipid metabolism to regulate the activity of stromal and immune cells, making them resistant to therapy and promoting disease recurrence. Due to the important role of ROS in the regulation of immunity, considering the connection between ROS and the regulation of lipid metabolism, we hypothesized that ROS might change the lipid composition in tumor cells and immune cells by regulating the activities of enzymes related to lipid metabolism and then affect the interaction between immune cells and tumor cells. Similarly, lipid metabolites may also modulate the redox state within the cell, affecting the level of ROS and thus exerting a regulatory effect on the immune response.

**Figure 4:**
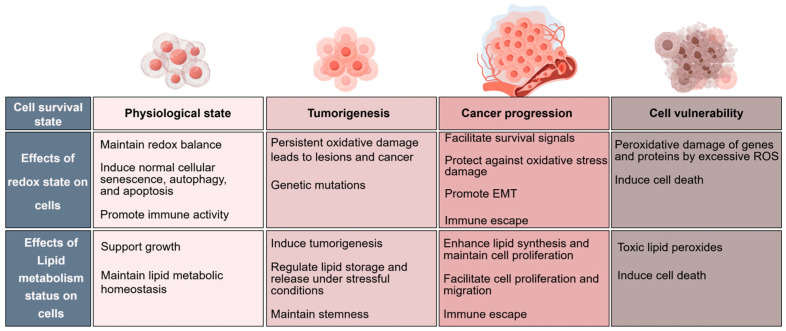
Effects of oxidative stress and lipid metabolism levels on cells. Oxidative stress signals and lipid metabolism levels maintain cell homeostasis and improve cell adaptability to the environment in the normal physiological environment. Under different circumstances, cellular responses to oxidative stress and lipid metabolism can either stimulate tumorigenesis and progression or induce cell death.

In nonmalignant diseases, the imbalance between the oxidative stress response and lipid metabolism is usually manifested as chronic inflammation, metabolic disorders, and tissue damage. In cancers, changes in oxidative stress and lipid metabolism are usually more complex. The abnormal lipid metabolism of cancer cells usually supports their high energy demand and rapid proliferation or increases their ability to tolerate oxidative stress through lipid metabolism. In conclusion, there are obvious differences in the roles of oxidative stress and lipid metabolism in nonmalignant diseases and cancers. In nonmalignant diseases, accumulating oxidative stress often leads to the progression of chronic diseases by damaging lipid metabolism; meanwhile, in tumors, oxidative stress and abnormal lipid metabolism jointly promote the proliferation and metastasis of cancer cells. The amplification or weakening of intracellular oxidative stress and abnormal lipid metabolism may lead to the vulnerability of cell viability and provide an entry point for treatment (Figure 4). Treatment strategies need to be tailored to the specific disease type, considering both the use of antioxidants, the regulation of lipid metabolism, and the induction of cell death by treatment. The effectiveness of lipid metabolism-targeted therapy may depend on the type of cancer, its metabolic characteristics, the tumor microenvironment, and its heterogeneity. For tumors that are highly dependent on lipid synthesis and metabolism, lipid metabolism-targeted therapies will be a promising treatment strategy. However, due to drug toxicity, drug resistance, and compensatory effects, strategies that address this process are rarely translated into clinical practice [46]. Combining lipid metabolism-targeted therapies with other treatment modalities may prove to be more effective. At present, remarkable progress has been made in the targeted therapy of cancer, especially in the fields of targeted drugs, immunotherapy, nanomedicine delivery, and gene editing. Studies have shown that, despite the challenges of, for example, drug-targeting accumulation efficiency, tumor heterogeneity, and drug resistance, issues such as the short half-life of siRNA and potential immune responses remain incompletely addressed. Nevertheless, with the advancement of technology and the state’s support for the research and development of innovative drugs, the number of targeted drugs approved in the Chinese market will gradually increase in the future, and precise targeted therapy remains the future direction of cancer treatment.

In summary, lipid oxidation and oxidative stress interact with each other and together play key roles in a variety of physiological and pathological processes. A deeper understanding of the relationship between them will help us to better recognize and address many diseases related to oxidative stress or abnormal lipid metabolism, as well as to interpret the occurrence and development of tumors.

## Figures and Tables

**Table 1:** Role of lipid metabolism-related genes in cancer.

Gene	Function	Regulation	Reference
FABP	Promotes cancer cell uptake and storage of lipids; enhances tumor proliferation and metastasis	PPAR,	[54,55]
ACSS	Sustains cancer cell growth in the absence of nutrients by ingesting and converting acetic acid	SREBP, deacetylation	[56]
SREBP1	Upregulates lipogenesis, promoting tumor growth	Ubiquitination, P53 mutation	[63,66,70,103]
FASN	Mediates lipid synthesis to promote tumor growth and invasion	SREBP, Acetylation	[63,68,70,84]
ACLY	Promotes lipid metabolism to support tumor growth	SREBP, Acetylation	[63,69,83]
ACSL1	FAO promotes tumor growth and maintains tumor stem cell-like characteristics	SREBP, methylation	[80,81]
ACSL4	Regulates fatty acid metabolism, maintains stemness, or promotes metastasis and invasion	c-Myc, methylation	[82,122]
SCD1	Monounsaturated fatty acids promote cell proliferation and enhance cell stemness	SREBP	[63,83]
MAGL	Supports membrane synthesis and generates a range of proto-oncogene signals through lipid hydrolysis	Unclear	[88]
ACAT1	Catalyzes the conversion of excess cholesterol to CEs; promotes tumor growth	SREBP, methylation	[92,93]
SOAT	Promotes cholesterol storage to support tumor growth	SREBP	[94,95,96]
ABCA1	Initiates the excessive activation of the lipid transport system to drive tumor growth	P53, methylation	[99,100,101]
HMGCR	Increases the rate of cholesterol biosynthesis to promote tumor growth	SREBP, deubiquitylation	[102]
ChoKα	Activates PC biosynthesis, promoting tumor progression, metastasis, and invasion	Unclear	[115]
LPCAT1	Increases saturated phosphatidylcholine content to promote plasma membrane synthesis and signal transduction	Unclear	[116]
G3P	Promotes glycerophospholipid synthesis and supports membrane synthesis and signal transduction	Unclear	[119]
